# A pilot registry of unexplained fatiguing illnesses and chronic fatigue syndrome

**DOI:** 10.1186/1756-0500-6-309

**Published:** 2013-08-02

**Authors:** Dana J Brimmer, Elizabeth Maloney, Rebecca Devlin, James F Jones, Roumiana Boneva, Caryn Nagler, Lisa LeRoy, Scott Royal, Hao Tian, Jin-Mann S Lin, Jennifer Kasten, Elizabeth R Unger

**Affiliations:** 1 Division of High-Consequence Pathogens and Pathology, Centers for Disease Control and Prevention, 1600 Clifton Road, MS-G41, Atlanta, GA 30033, USA; 2 Abt SRBI, 640 North LaSalle, Suite 640, Chicago, IL 60610, USA; 3 McKing Consulting, 2900 Chamblee Tucker Road, Building 10, Suite 100, Atlanta, GA 30341, USA; 4 Abt Associates, 55 Wheeler Street, Cambridge, MA 02138-1168, USA; 5 Current Affiliation: Food and Drug Administration, Office of Surveillance and Epidemiology, 10903 New Hampshire Ave., Building 22, Rm 2476, Mail Stop 3411, Silver Spring, MD 20993, USA; 6 Current Affiliation: JBS International, Inc., 5515 Security Lane, Suite 800, North Bethesda, MA 20852, USA

**Keywords:** Chronic fatigue syndrome (CFS), Fatiguing illness, Registry, Education

## Abstract

**Background:**

Chronic fatigue syndrome (CFS) has no diagnostic clinical signs or biomarkers, so diagnosis requires ruling out conditions with similar signs and symptoms. We conducted a pilot registry of unexplained fatiguing illnesses and CFS to determine the feasibility of establishing and operating a registry and implementing an education outreach initiative. The pilot registry was conducted in Bibb County, Georgia. Patient referrals were obtained from healthcare providers who were identified by using various education outreach initiatives. These referrals were later supplemented with self-referrals by members of a local CFS support group. All patients meeting referral criteria were invited to participate in a screening interview to determine eligibility. If patients met registry criteria, they were invited to a one-day clinic for physical and laboratory evaluations. We classified patients based on the 1994 case definition.

**Results:**

We registered 827 healthcare providers. Forty-two providers referred 88 patients, and 58 patients (66%) completed clinical evaluation. Of the 188 CFS support group members, 53 were self-referred and 46 (87%) completed the clinical evaluation. Of the 104 participants completing evaluation, 36% (n = 37) met the criteria for CFS, 17% (n = 18) had insufficient fatigue or symptoms (ISF), and 47% (n = 49) were found to have exclusionary medical or psychiatric illnesses. Classification varied significantly by type of referral but not by previous history of CFS diagnosis. Healthcare providers referred more patients who were classified as CFS as compared to support group referrals in which more exclusionary conditions were identified. Family practice and internal medicine specialties made the most referrals and had the highest number of CFS cases. We conducted three CME events, held three “Meet and Greet” sessions, visited four large clinical health practices and health departments, mailed five registry newsletters, and conducted in-person office visits as part of education outreach, which contributed to patient referrals.

**Conclusions:**

Referrals from healthcare providers and self-referrals from the patient support group were important to registry enrollment. The number of potentially treatable conditions that were identified highlights the need for continued medical management in this population, as well as the limitations of registries formed without clinical examination. Education initiatives were successful in part because of partnerships with local organizations.

## Background

Chronic fatigue syndrome (CFS) is a debilitating condition that affects over 1 million adults in the United States with many more experiencing chronic symptoms of fatigue [1-4]. A CFS diagnosis is based on self-reported chronic symptoms, unexplained severe fatigue present for six months or more, and at least four of eight other symptoms (impaired memory or concentration, muscle pain, pain in multiple joints, unrefreshing sleep, tender lymph nodes, sore throat, headaches, post-exertional malaise) that are severe enough to significantly interfere with the performance of daily activities and work [5]. Diagnosis of CFS requires ruling out concurrent medical and psychiatric conditions with similar clinical signs and symptoms. CFS has no diagnostic biomarkers, signs, or laboratory tests, and despite over two decades of research, the etiology and pathophysiology are yet to be determined.

The lack of objective diagnostic criteria poses a challenge for physicians and helps explain the low diagnostic confidence [6]. In spite of this, most physicians (94%) are aware of CFS, and 40% report having diagnosed the illness [7]. This high level of provider awareness belies estimates that fewer than 20% of people with CFS have been diagnosed [8].

CFS patient registries can provide information concerning the nature of the illness among those receiving medical care. Twin registries and the Gulf War Registry have been used to study CFS prevalence, the medical impact of illness, and the role of environmental and genetic factors [9-13]. However, published twin registries have not medically evaluated participants. They use questionnaires to assess and classify fatigue and CFS often in conjunction with reported physician diagnosis [12,13]. Registries, such as the United Kingdom pilot CFS/ME registry, also provide a cost effective means of evaluating aspects of CFS, identifying CFS for clinical studies, and evaluating clinical course [14]. In conjunction with population-based CFS surveillance, registries provide a means of exploring factors associated with health care use by people with CFS.

This report concerns the feasibility of establishing and operating a pilot registry that was conducted in Bibb County, Georgia. The objectives of this registry were 1) to recruit and register medical and ancillary healthcare providers in and around Bibb County, 2) to encourage enrolled healthcare providers to refer patients with unexplained fatiguing illnesses and CFS, 3) to clinically evaluate referred patients, and 4) to implement an education outreach initiative. The registry was later supplemented by local support group members through self-referral. We clinically evaluated all referred patients according to the protocol used in population surveillance so as to diagnose CFS by international criteria [5], identify exclusionary medical and psychiatric conditions, and collect sociodemographic and related information.

## Methods

The pilot registry was conducted in Bibb County, Georgia from September 2008 to March 2010. This pilot included a six-month healthcare provider recruitment phase followed by a full year of patient referrals to a research clinic, where patients were clinically evaluated [1]. A needs assessment in the form of focus groups with Bibb County healthcare providers was conducted before the registry, and these results helped to inform the registry study design. For example, time commitments and burden on support staff were identified as concerns, whereas a benefit was the desire to receive updates throughout the study period. In December 2009 the Registry expanded to allow members of the local CFS support group to self-refer to the registry. This study adhered to U.S. Department of Health and Human Services policies for the protection of human research subjects and was approved by the CDC Institutional Review Board. All participants gave informed consent. The registry methods are reported in five parts: Healthcare Provider Recruitment, Healthcare Provider Enrollment and Referrals, Support Group Referral, Referrals for Clinical Evaluation, and Registry Education Initiatives.

### Healthcare provider recruitment

Healthcare providers included traditional medical providers (e.g., physicians and nurse practitioners) as well as complementary and alternative medical providers (e.g., acupuncturists and physical therapists) (see Additional file 1: Table S1 for a full list of healthcare provider categories by physicians and non-physicians). We identified healthcare providers in and around Bibb County, Georgia through data obtained from insurance websites, a purchased external vendor list, the Georgia State Board of Medical Examiners, Internet searches of local medical practices, and from a local support group.

We aimed to recruit 600 healthcare providers who would in turn refer patients for registry enrollment and clinical evaluation. Eligibility criteria for healthcare providers included: currently practicing in Bibb County or within a 30-mile radius of Bibb County, and willingness to assess patients for registry inclusion/exclusion criteria. Exclusion criteria for healthcare providers included practicing exclusively in a nursing home, Veteran’s Affairs (VA) hospital, mental health hospital, military or correctional facility; being retired or deceased; and not currently treating or diagnosing patients.

In early August 2008, we sent a one-page flier announcing the registry to the 1,787 identified healthcare providers and invited them to attend a CDC-sponsored CFS continuing medical education (CME) course. In early September 2008, a Registry Kick-Off packet was sent to all identified providers, which included a registry introduction letter, registry fact sheet, and an enrollment form. In November 2008, a second mailing was sent that included the incentive of a $1 bill to increase attention and awareness. In December 2008, a local Georgia-based firm was contracted to actively recruit and register providers by calling and visiting offices and track provider recruitment and enrollment status. This firm added a proprietary marketing list to the database for a final master list of 2,190 healthcare providers. Provider recruitment outcomes were classified as verified eligible (eligible providers who were enrolled in the registry); ineligible (providers who did not meet inclusion criteria); refused (providers who refused to participate); unlocatable (providers who could not be reached at listed contact information); and nonresponse (providers who were not reached within 5 telephone contacts).

### Healthcare provider enrollment and referrals

Healthcare providers were sent a Provider Verification Form for assessment of inclusion and exclusion criteria; providers were requested to return the form by fax, mail with pre-paid envelope, phone call to the toll-free registry number, or use of the registry website. Once providers were registered, they were sent a Provider Packet and Patient Referral Packets. The Provider Packet contained a letter with a unique provider code; login and password for the registry website, a provider referral form; registry referral criteria (Table 1); a questionnaire for measuring knowledge, attitudes, and beliefs about CFS; and a fact sheet of responses to frequently asked questions. The provider was instructed to give the Patient Referral Packet to eligible patients. The Patient Referral Packet contained a letter for the adult or parents of adolescent participants, a permission-to-be-contacted form, and a fact sheet about the registry and questions frequently asked by patients.

**Table 1:** Registry referral inclusion and exclusion criteria

Inclusion referral criteria*	Exclusion referral criteria*
To be eligible for referral to the registry, an individual must have met the following criteria:	Providers were instructed to rule out other potential causes of fatigue by conducting laboratory tests and medical history or medical histories alone before referring their patients. The registry had the following exclusion criteria:
• Unexplained severe fatigue persisting for one month or longer.	
• One of the following for at least one month:	
o unrefreshing sleep,	• Pregnancy within past 12 months
o problems with memory or concentration, or	• Stroke with no full recovery
o unexplained joint or muscle pain.	• Parkinson’s disease
• A body mass index of less than 40.0	• Chronic obstructive pulmonary disease (COPD) or congestive heart failure
• Age 12 to 59 (However, in November 2009, CDC expanded the eligible age range to 69 years of age. This upper age range limit was clarified to mean that in order to be eligible, the subject must be no more than 69 years of age as of the date of the most recent CDC IRB approval, September 2009).	
	• Insulin-dependent diabetes
	• Uncontrolled diabetes type II (HgbA1c < 9%)
	• Anemia
	• Uncontrolled hypo- or hyper-thyroidism
	• Uncontrolled hypertension (BP > 140/90)
	• Sickle cell anemia
	• Cancer within 5 years (except basal skin) or current chemotherapy
	• Untreated depression
	• Substance abuse problems within the past 2 years
	• Anorexia nervosa or bulimia nervosa within the past 5 years
	• Schizophrenia, bipolar I or II disorder, or dementia
	• Hepatitis B or C

*The registry inclusion/exclusion referral criteria are not for CFS classification.

In making a referral, healthcare providers assessed patients only for registry referral criteria; not for chronic fatigue syndrome. Inclusion criteria for patients consisted of medically unexplained, severe fatigue persisting for one month or longer and at least one month’s duration of sleep, or problems with memory or concentration, or unexplained joint or muscle pain; a body mass index less than 40; and age 12–69 as of September 15, 2009 (Table 1). Exclusion criteria noted in Table 1 are consistent with the 1994 CFS case definition [5] (Table 1). Registry referral criteria were deliberately relaxed to capture patients who would be further assessed for CFS later during clinical evaluation at the registry clinic. Because a differential diagnosis of fatigue can take months, providers did not have to conduct a full workup of CFS. Instead, physicians were asked to conduct basic blood tests in addition to a medical history, and non-physicians were required to take a medical history.

Registered health care providers filled out the provider referral form and asked their patients to fill out the “permission-to-be-contacted form” if they were interested in participating in the registry. Patients could not be contacted by registry staff if the completed permission form was not received, even if the provider had submitted the provider referral form. When this situation occurred, a two-week follow-up courtesy call was made to the referring provider to notify him or her about the missing permission form from the patient, with the hope that the provider would remind the patient. Patients who never submitted their permission forms were ultimately coded as nonresponders. Providers were instructed to return their completed referral forms by fax, mail, toll free number, or the registry website. Patients could return permission-to-be-contacted forms by fax, mail, or toll free number.

We used several strategies to encourage healthcare providers to refer patients. Registry staff made in-person visits to large medical practices (those with at least 10 providers at a single site) and to providers who had been registered for at least three months without making any referrals. Visits to large medical practices included lunch and a brief presentation on the registry referral criteria and process; visits to individual providers included distribution of extra materials and in some cases, an appreciation gift of either M&Ms or a pen. Additionally, providers were sent a thank you note upon the referral of each patient.

### Support group referral

CDC partnered with a local support group early in the registry to apprise them of the registry’s research objectives, although referrals from support group members were not solicited in the first year of patient enrollment. The local support group was founded in 1990 and has met once a month since its inception; it serves as a safe place for people to talk about their illness and gain acceptance as well as obtain educational information. Membership has no requirements; however, most members have been diagnosed with CFS or fibromyalgia. In October 2009, with the backing of the support group leader, we gave a more in-depth presentation of the registry goals and invited support group members to refer themselves to the registry staff. Support group members (n = 188) were sent packets that included an instruction letter describing how to contact the registry, inclusion and exclusion criteria (Table 1), frequently asked questions, and a body mass index (BMI) table for determining whether their BMI exceeded the inclusionary range. Support group members were expected to review the inclusion and exclusion criteria and determine whether they met criteria for self-referral; if so, members could refer themselves to the registry by using the toll free number.

### Referrals for clinical evaluation

Upon receipt of the permission-to-be-contacted form (provider-referred patients) or the self-referral phone call (support group members), registry staff contacted prospective registry patients for a screening interview. The screening interview was conducted by using a computer-assisted telephone interview (CATI) from September 2008 through November 2009. CDC physicians and study personnel reviewed all CATI data and determined final clinical eligibility. Patients meeting screening criteria were then contacted to arrange an appointment for their clinical evaluation. Given the high eligibility rate, we converted the CATI to a computer-assisted personal interview (CAPI) in December 2009. The CAPI screening questions were identical to items on the CATI, but CAPI was interviewer-administered onsite in the registry clinic. Patients who did not complete the CATI received a short screening call before clinic scheduling to determine fatigue and symptom eligibility status.

All patients (provider- and self-referred) were evaluated by the same registry referral inclusion and exclusion criteria. For example, for patients who responded during screening that they experienced fatigue that lasted less than six months, a registry staff member re-contacted the person to notify them of their temporary ineligibility status as well to provide a date for call-back (e.g., reassess fatigue at the six-month mark).

Patients meeting eligibility criteria were invited to a one-day clinical evaluation at a local research clinic and were sent a packet of materials before the appointment [1]. The packet included an introductory letter, sample clinic schedule, informed consent form for review, and registry questionnaires to complete at home. Additionally, patients received materials and instructions on how to prepare for their clinic visit, including fasting the morning of the clinic, foregoing vitamins, herbal or nutritional supplements, and homeopathic preparations for 72 hours before the clinic date, collecting and storing a 12-hour urine specimen (night before clinic visit) and collecting waking saliva samples (morning of clinic visit). The clinical evaluation took approximately eight hours to complete, and patients were provided with a $450 reimbursement for their time. Informed consent was administered by registry staff on the day of the appointment.

The clinical assessment included medical history, a standardized complete physical exam, collection of urine and blood for laboratory tests, and psychiatric evaluation by using the Structured Clinical Interview for DSM Disorders (SCID) [15]. Clinical assessment also ascertained demographics, symptoms, various risk factors, and medication use. To ensure uniform practice, clinic staff (including physicians) received eight hours of instruction on conducting a standardized clinical examination and collecting a medical history. All clinic staff underwent a training session to complete intake materials and administer questionnaires. After the clinical evaluation and upon receipt of registry clinic data, CDC physicians reviewed all medical history questionnaires, laboratory test results, and clinical evaluation data to determine exclusionary medical or psychiatric conditions. Patients were classified into three categories based on the 1994 CFS case definition [5]: chronic fatigue syndrome (CFS); insufficient fatigue or number of symptoms to meet the CFS definition (ISF); or having an exclusionary condition, and therefore not qualifying for the 1994 Case Definition.

### Registry education initiatives

An important component of the registry was to provide medical education to registry healthcare providers and the local healthcare community. CDC partnered with a local medical society to raise awareness and encourage participation in the registry. CME and “Meet and Greet” events were co-sponsored by the local medical society and the local university. CME events were held at the local hospital, local registry clinic, and university outpatient clinic. “Meet and Greet” events were held at the local registry clinic site, and local healthcare providers were invited to meet CDC and registry staff to learn more about the registry. Registry newsletters were produced and mailed to enrolled healthcare providers, which gave updates about recruitment and enrollment numbers, had an “Ask the CDC CFS Expert” column, cited newly published CFS journal articles, and provided location and contact information for CME and “Meet and Greet” events. Finally, on the basis of feedback from the needs assessment, we developed a website, which offered links for obtaining registry materials, registry forms, and CFS educational information.

## Results

### Healthcare provider enrollment and referrals

Eight hundred twenty-seven healthcare providers registered for the registry, including 491 (59%) physicians and 336 (41%) non-physicians for a response rate of 53% (Table 2). Fifty-five percent of the 491 enrolled physicians were family practice/internal medicine, 11% pediatric, and 10% obstetrics/gynecology. Of the 336 non-physicians enrolled in the registry, 39% were nurse practitioners, 18% dentists, and 12% registered nurses. Of registered providers, 38 had participated in needs assessment focus groups. Most providers enrolled through telephone recruitment (n = 779), followed by a second mailing, which included $1 incentive (n = 33); initial mailings (n = 6); provider initiated contact (n = 4); CME events (n = 3); and the registry website (n = 2).

**Table 2:** Provider recruitment by type of provider (physician vs. non-physician)

	**Verified eligible**	**Ineligible**	**Refused**	**Unlocatable**	**Non response**	**Total**
	**n (%)**	**n (%)**	**n (%)**	**n (%)**	**n (%)**	**n (%)**
**Physicians**	491 (59)	147 (81)	59 (59)	373 (83)	99 (16)	1169 (57)
**Non-physicians**	336 (41)	33 (18)	41 (41)	75 (17)	532 (84)	1017 (46)
**Other/Unidentified**^**a**^	0	1 (<1)	0	1 (<1)	2 (<1)	4 (<1)
**Total**	827	181	100	449	633	2190

^a^Other/Unidentified refers to providers for whom the specialty is not known, with the exception of the “other” Ineligible, who was an office manager.

Forty-two enrolled healthcare providers referred 88 patients to the registry. Sixty-four percent of all referrals came from physicians (n = 56) and 36% from non-physician providers (n = 32). Nineteen providers referred more than one patient, including one provider who referred nine patients. Physicians who referred patients included family/general practitioners (n = 16), internists (n = 15), pediatricians (n = 10), obstetrics and gynecologists (n = 9), geriatricians (n = 3), pain medicine specialists (n = 1), and psychiatrists (n = 2). Non-physician providers who referred patients were psychologists (n = 13), nurse practitioners (n = 8), dentists (n = 7), an acupuncturist (n = 1), a social worker (n = 1), a physical therapist (n = 1), and a massage therapist (n = 1).

Healthcare providers’ preferred methods of returning the provider referral form were fax (n = 35) or mail (n = 33). Patients’ preferred methods included mail (n = 38), fax (n = 24), and phone (n = 15). Although 88 patients were referred for clinical evaluation, registry staff could only contact 78 because 10 people did not return the permission-to-be-contacted form. Thus, 11% of referrals were automatically excluded from potential clinical evaluation.

### Support group referral

Fifty-three (28%) of 188 support group members referred themselves to the registry. Six study packets to support group members were returned with no forwarding address for a response rate of 29% (53/182). Support group referrals accounted for 38% of all registry participants.

### Referrals for clinical evaluation

Twelve (15%) of 78 patients referred by providers were found ineligible at the time of pre-clinic screening. Only two (4%) of 53 self-referred patients were ineligible at pre-clinic screening (Table 3). Five people refused to participate in the clinical evaluation and eight never responded to the invitation for clinical evaluation. Thus, 104 of 131 referred patients completed clinical evaluation for a 79% response rate. (See Additional file 2: Figure S1 for a flow chart of provider recruitment and patient enrollment).

**Table 3:** Referrals for clinical evaluation

	**Complete**	**Nonresponse**	**Refusal**	**Ineligible**	**Total**
	**n (%)**	**n (%)**	**n (%)**	**n (%)**	**n (%)**
**Provider-referred patients**	58 (56)	4^b^ (50)	4 (80)	12^c^ (86)	78 (60)
**Support group self-referrals**	46^a^ (44)	4^d^ (50)	1 (20)	2^e^ (14)	53 (40)
**Total**	104	8	5	14	131

^a^One patient signed an informed consent form agreeing to share data from the GA First Follow-up Study with the GA Registry.

^b^Non-contact (n = 1); moved out of area (n = 1); unlocatable (n = 2).

^c^Ineligible baseline registry subject (n = 1); ineligible based on age (n = 2); duplicate -- referred by 2 providers (n = 1); duplicate - referred under two different names (married and unmarried) (n = 1); Bipolar disorder reported during CATI interview (n = 2); BMI > 40 (n = 3); Rheumatoid arthritis reported during CATI interview (n = 1); BMI > 40 and emphysema reported during CATI interview (n = 1).

^d^Appointment canceled, had “too much going on” (n = 1), not feeling well enough to participate (n = 2); unlocatable (n = 1).

^e^BMI > 40.

### Registry education and outreach initiatives

Over the pilot registry time period, we held three local CME events, sponsored three “Meet and Greet” sessions, published and distributed five newsletters to all 827 enrolled providers and maintained the registry website. Ninety-six people received CME credits and 25 providers attended the “Meet and Greet” sessions. The registry website received more than 1700 visits, 87% (n = 1517) from within the United States. Only two providers registered through the website, and no providers referred patients through the site. Outreach visits to healthcare providers were conducted to stimulate participation in the registry. Visits were made to 19% (n = 157) of enrolled providers; some received a visit only (n = 87) and some received a visit with an appreciation gift (n = 70). Four visits were also made to large clinical practices in Bibb County and the local health department.

Overall, 62% (n = 26) of providers who made referrals (in some case multiple referrals) had either participated in the focus groups, a CME session, “Meet and Greet” event, or had received a visit. Seventy-four percent (n = 65) of all patient referrals and 66% (n = 38) of clinical evaluation patients were by providers who participated in an education or outreach activity of some type (Table 4).

**Table 4:** **Impact of education and outreach activities on referrals and clinic evaluation**^**1**^

**Activity**	**# of providers**	**# of referrals**	**# of clinic evaluations**
	**n (%)**	**n (%)**	**n (%)**
Focus group	2 (5)	10 (11)	5 (9)
CME^a^	3 (7)	4 (5)	3 (5)
Meet and greet	3 (7)	7 (8)	3 (5)
Incentive visit	4 (10)	12 (14)	6 (10)
Visit only	13 (31)	31 (35)	20 (34)
Two activities	1 (2)	1 (1)	1 (2)
No contact	16 (38)	23 (26)	20 (34)
**Total**	42	88	58

^a^Continuing medical education.

^1^Note: 36 (5%) of the 785 enrolled providers who did not make any referrals participated in the focus group, 66 (8%) had incentive visit, 73 (9%) had visit only, and 22 (3%) attended Meet and Greet (data on CME not available).

### Clinical evaluation results

A total of 104 patients completed clinical evaluations, including 5 adolescents (Table 5). On the basis of the 1994 case definition [5], 36% (n = 37) of patients had CFS, 17% (n = 18) ISF, and 47% (n = 49) had an exclusionary medical or psychiatric diagnosis. The sample was predominately female (89%, n = 93), white (88%, n = 91), and well-educated; 88% (n = 92) had a high school degree or above. Mean age was 47.4 (SD = 14.6) (range 13 – 70), and mean BMI was 27.2 (SD = 5.4). Forty-four percent (n = 46) of those evaluated in the clinic were self-referred support group members, and 58% (n = 57) (excluding adolescents) reported receiving a prior CFS diagnosis before participation in this registry.

**Table 5:** Demographics of clinical sample by illness classification

	**CFS**	**ISF**	**Exclusion condition**	**Total**
	**n (%)**	**n (%)**	**n (%)**	**n (%)**
**Sex**				
Female	33 (89)	13 (72)	47 (96)	93 (89)
Male	4 (11)	5 (28)	2 (4)	11 (11)
**Age**				
Adolescent (<18)	1 (3)	3 (16)	1 (2)	5 (5)
18-20	0	0	3 (6)	3 (3)
21-30	3 (8)	3 (16)	2 (4)	8 (8)
31-40	9 (24)	3 (16)	5 (11)	17 (16)
41-50	6 (16)	1 (6)	8 (16)	15 (14)
51-60	12 (32)	6 (33)	19 (39)	37 (36)
61-70	6 (16)	2 (11)	11 (22)	19 (18)
**Race**				
White	33 (89)	18 (100)	40 (82)	91 (88)
Black	3 (8)	0	7 (14)	10 (10)
Other	1 (3)	0	2 (4)	3 (3)
**Education**				
1st through 8th grade	0	2 (11)	0	2 (2)
Some high school	3 (8)	2 (11)	5 (10)	10 (10)
High school graduate	5 (13)	1 (6)	8 (16)	14 (13)
Trade/Technical/Vocational after				
High School	3 (8)	0	9 (18)	12 (12)
Some college	6 (16)	3 (17)	13 (27)	22 (21)
Two-year college graduate	6 (16)	4 (22)	5 (10)	15 (14)
Four-year college graduate	6 (16)	4 (22)	2 (4)	12 (12)
Postgraduate	6 (16)	2 (11)	5 (10)	13 (12)
Other	2 (5)	0	2 (4)	4 (4)
**BMI**				
≤18	2 (5)	2 (11)	0	4 (4)
19-24	12 (32)	5 (26)	15 (31)	32 (31)
25-30	16 (43)	7 (58)	21 (43)	44 (42)
31-39	8 (22)	4 (21)	13 (27)	25 (24)
**Previous CFS Diagnosis**^**a**^				
Yes	20 (54)	10 (56)	27 (56)	57 (55)
No	16 (43)	5 (28)	21 (44)	42 (40)
**Total**	37	18	49	104

^a^Excludes adolescent patients (n = 5).

Of the 49 people classified as having an exclusionary condition, the five most common exclusionary conditions were hypothyroidism, diabetes, alcohol abuse, anemia, and high levels of C-reactive protein (see Table 6 for a complete list).

**Table 6:** **Exclusion conditions at clinical evaluation**^**a**^

**Exclusion condition**	**Support group referral**	**Provider referral**
	**(n = 29)**	**(n = 20)**
Active inflammation	1	1
Alcohol abuse	4	–
Anemia	2	1
Anorexia bulimia	–	1
Autoimmune disorder	1	–
Bipolar	–	2
Cervical, thoracic, lumber spine disease	–	1
Diabetes Type II/insulin resistance	7	1
Hepatitis C	1	–
High blood urea	2	–
High C-reactive protein (CRP)^a,b^	5	5
Hypertension	1	–
Hypothyroidism	7	3
Major depressive disorder with melancholy	2	2
Mitochrondrial myopathy	–	1
Obesity	2	–
Obstructive sleep apnea	1	1
Osteoarthritis	2	–
Narcolepsy	1	–
Restless leg syndrome	2	1
Rheumatoid arthritis	1	–
Sleep problems	1	–
Schizophrenia	1	–
Sickle cell	–	1
Substance abuse	–	3
Uncontrolled high blood pressure	1	–
Urinary tract infection	2	2
**Total exclusion condition by group**	**47**	**26**

^a^Person could have more than one exclusion condition and the exclusion could be based on one or multiple conditions.

^b^All persons with high CRP were excluded for other coexisting medical conditions. While a high CRP value is not an exclusion as defined in the 1994 case definition, we identified persons with CRP values that were several fold higher than the upper limits of normal values, as there may be an underlying inflammatory, infectious, or cardiovascular disease.

Sixty-five percent (n = 24) of provider-referred patients were CFS cases as compared to 35% (n = 13) of support group referrals. Fifty-nine percent (n = 29) of support group referrals had exclusionary conditions compared to 41% (n = 20) of provider-referred patients. Healthcare providers referred 14 people (78%) who were classified as ISF as compared to 4 (22%) support group referrals. Illness classification varied significantly by type of referral method; support group referrals had the lowest proportion of patients classified as ISF but the highest proportion of exclusions (x^2^ = 9.21, p < 0.05) (Table 7). No significant association was found between history of CFS diagnosis and illness classification. Clinical outcome categories did not differ between the physician and non-physician groups.

**Table 7:** Illness classification by referral and previous CFS diagnosis n (%)

	**CFS**	**ISF**	**Exclusion condition**	**χ**^**2**^
	**n = 37**	**n = 18**	**n = 49**	
**Referral method**				
Provider	24 (65)	14 (78)	20 (41)	9.21*
Support group	13 (35)	4 (22)	29 (59)	
**Previous CFS Diagnosis**^**a**^				
Yes	20 (56)	10 (67)	27 (56)	7.25
No	16 (44)	5 (33)	21 (44)	

*p < 0.05.

^a^Excludes adolescent patients (n = 5).

## Discussion

The pilot registry exceeded the recruitment goal of 600 by registering 827 healthcare providers. Despite multiple mailings, CME events and a registry website, telephone recruitment was the most successful method for provider registration, accounting for 94% of provider registration. One reason that telephone recruitment worked well is that healthcare providers could immediately respond to verification questions upon receipt of the phone call. Focus groups had suggested that a registry website would be desirable for provider registration; nevertheless, only 2 of 827 providers registered through the site. However, enrolling through the website, fax, or postal mail required a two-step process: 1) filling out the Provider Verification Form and 2) submitting the information. Given time constraints and other practice priorities, providers may have been dissuaded to register through these channels.

Despite success in recruiting healthcare providers, only 42 (5%) of the 827 enrolled providers referred patients for clinical evaluation, even though referral criteria were relaxed and based on fatiguing illnesses and not just CFS (Table 1). Several explanations may account for this outcome. First, healthcare providers were asked to refer patients that came into their offices during the study enrollment period and not to look in medical charts for referrals, thereby missing patients who met inclusion criteria. Second, we opened registry registration to healthcare providers of all specialties and not just to those who see or treat people with fatigue. Many specialty providers may not have had the opportunity for referral, as their patient population would not be seeking care for fatigue. Supporting this concept is the fact that none of the enrolled healthcare providers from anesthesiology, allergy and immunology, infectious disease, rheumatology, endocrinology, gastroenterology, neurology, surgery, orthopedics, or ophthalmology made referrals, whereas primary care providers (family practice and internal medicine) generated the greatest number of referrals (n = 33, 38%). This finding also supports the view that many people with unexplained fatiguing illnesses continue to seek healthcare from their primary care providers. Finally, the firm hired to recruit providers was compensated per enrolled provider and therefore had an incentive to recruit and enroll as many providers as possible, including providers who may not have had an interest in chronic fatigue or the registry.

Of the 42 providers who made referrals, 62% received an outreach visit or attended either a focus group, CME, or “Meet and Greet” event. Of healthcare providers who received education or outreach, three-quarters referred patients and two-thirds had patients complete the clinical evaluation. Thus, education and active outreach to providers may have contributed to a greater number of patient referrals and clinic evaluations as compared to no participation or providers who did not receive these initiatives. When comparing education events (focus groups, CME, or “Meet and Greet”) to outreach activities (visits or visits with incentives), outreach resulted in a higher number of referrals (49%) and clinic evaluations (44%) compared to education. Researchers interested in developing and operating a registry in which referrals are a goal may want to consider incorporating visits to healthcare providers as one technique to increase patient referral rates.

We opened registry enrollment to local support group members. Although the self-referral process worked well, with no complaints or confusion, only 28% of the support group referred themselves for screening. Despite the need for further research, results from this pilot registry suggest that future CFS registries may want to consider including enrollment directly from patient populations if full clinical and laboratory exams are included in a clinical assessment.

One-hundred and four patients were clinically evaluated and, of these patients, 37 had CFS, 18 ISF, and 49 an exclusionary condition. A little more than half of the sample reported a previous CFS diagnosis, but this history was equally distributed across illness classification conditions. A relatively high proportion (47%) of the registry patients had exclusionary conditions identified after clinical evaluation. This finding replicates other CFS studies in which 50 - 70% of patients who met symptom criteria were found to have medical or psychiatric exclusions [3,16,17]. Future research needs to examine the potential for detecting treatable medical exclusionary conditions among people suffering from fatiguing illnesses because treatment may improve fatigue and associated symptoms for many of these patients.

More revealing was the proportion (59%) of exclusions found in the support group as compared to the provider referred group. In fact, 63% of the support group patients evaluated (29/46) had an exclusionary condition identified. Despite awareness of their illness (as evidenced by membership in support group), the self-referral group had undetected medical conditions, and some patients experienced more than one exclusionary condition. For example, this group had seven cases of diabetes compared to one in the provider referral group, and seven cases of hypothyroidism compared to three in the provider group. In both groups, many of the exclusionary conditions were detected on the screening laboratory tests and are treatable.

Forty-one percent of provider referrals were determined to have an exclusionary condition. Because providers were given a medical exclusion list and knew the medical history of patients, one would expect lower exclusion rates, e.g., few provider referrals resulting in exclusions. Two possible explanations address this phenomenon. First, most of the exclusions were identified on the basis of laboratory tests. Physician providers were asked to give potential referrals by running basic lab tests and completing a medical history; however, referrals could be made without the requirement for a complete work-up, and laboratory testing may not have been conducted. Lack of basic lab tests may have been especially true for non-physician referrals, for which the requirement was a medical history. Second, providers may have welcomed participation in the registry as a method to get assistance for diagnosing difficult or unknown etiologies. Finally, the other main category of exclusions for provider referrals were bipolar disorder, major depressive disorder, substance abuse, and anorexia bulimia, which is understandable as some of these diagnoses are difficult to establish.

Sixty-five percent of provider-referred patients had CFS compared to 35% of support group referrals. Sixty-five percent of referred patients had CFS, which replicates similar findings from the CFS Rochester Epidemiology Project in which 66% of patients were accurately coded into the CFS category [17]. For an illness that has no known etiology or biomarkers, a fairly high level of knowledge and awareness was shown for providers who referred two-thirds of patients diagnosed with CFS at clinical evaluation. Although further research is warranted, part of the awareness may be due to the education and outreach events in which 62% providers participated. The main reason for not meeting CFS criteria was exclusionary conditions as opposed to being classified as ISF. A higher proportion of provider-referred patients (78%) were classified as ISF as compared to support group patients (22%). After exclusionary conditions are fully treated, those patients otherwise meeting criteria for CFS would be managed in the same way as CFS.

We aimed to increase awareness and knowledge of CFS among the healthcare community through several education initiatives. CME events offered the opportunity for professional credits and were offered through local partnerships. We found from anecdotal feedback that the newsletter, an item identified through the needs assessment, was well-received and appreciated by providers. As community-based research has shown [18], one of the most valuable aspects of this registry, which contributed to its success, was the partnership with the local medical society. The partnership allowed for educational outreach events with CME that would otherwise have been time consuming and difficult to implement. The partnership also gave registry staff access to the healthcare community and enabled us to participate with the community in building the registry.

This research has several limitations. The results reflect the experience of one study and future fatigue and CFS registries may have different recommendations, as registries have different study designs. The total number of patients evaluated (104) is relatively small and the pilot was conducted in one locality, so the findings may not be generalizable. We did not have a way to determine the number of potentially eligible patients that each provider saw during the time of the study. Because this study was a pilot registry, we did not determine if longitudinal information could be obtained.

While we did not exclude severely ill patients from study participation, the study design made it difficult for home-bound or other severely ill patients to participate as they would need to travel to the local clinic for the 8-hour testing day. In fact, two referred patients canceled their clinical evaluations because they were not feeling well enough to participate (Table 3). Researchers may want to consider this aspect of participant recruitment for future registries. Extra costs or other methodological issues may be incurred in arranging for testing at a patient’s house, but this would allow for home-bound and severely ill patients to be included in the registry.

Finally, depending on recruitment methods, cost and sustainability of registries are an important aspect of further research. For example, this study demonstrated that in the Bibb County, Georgia area telephone recruitment was the best method to enroll providers. We used several different methods, for example, mailing materials and a study website, before turning to telephone recruitment even though focus group results indicated recruitment by website was a preferred option. When developing future registries, researchers may want to consider various recruitment methods in order to sustain enrollment. All studies incur costs in the form of laboratory testing, medical staff time, and data collection; study objectives should be prioritized in terms of cost and sustainability.

In the needs assessment phase, providers had expressed concerns about the 1996 Health Insurance Portability and Accountability Act (HIPAA) procedures and the preference for not being involved in the process of consenting patients. These concerns proved challenging in implementing a system in which providers screened potential patients, but the patients had to complete the referral process. From the provider referrals, the registry staff could not contact 11% of patients for clinical assessment because 10 people did not follow through by submitting permission-to-be-contacted forms. This finding may also serve as a challenge to other registries that rely on physician referrals or access to medical records to confirm a diagnosis.

## Conclusions

A pilot registry of unexplained fatigue and CFS in which healthcare providers were registered to refer patients suspected of having unexplained fatiguing illness or CFS for a standardized clinical evaluation and diagnosis, provided information on clinicians’ accuracy in recognizing aspects of fatiguing illness and CFS. It also identified gaps in their knowledge, which may inform educational initiatives and ultimately increase the number of people receiving treatment. This pilot registry design allowed many different types of healthcare providers to participate and permitted tracking of medical specialty in terms of the referral process. The registry demonstrated the feasibility of including both provider and support group referrals. Results highlight the need and importance for inclusion of thorough clinical and laboratory exams in a CFS registry rather than relying on report of a previous CFS diagnosis. Many exclusion conditions were detected by laboratory tests and appeared to be unknown to the participating patients and healthcare providers. The number of untreated conditions in this population also emphasizes the need for continued medical follow-up to identify and manage conditions that may develop, rather than attributing all illness symptoms to CFS. Future CFS registries should consider how to address exclusions when enrolling potential patients.

## Abbreviations

CFS: Chronic fatigue syndrome; CDC: Centers for Disease Control and Prevention; ISF: Insufficient fatigue or symptoms; VA: Veteran’s Affairs; CME: Continuing medical education; CATI: Computer-assisted telephone interview; CAPI: Computer-assisted personal interview; BMI: Body mass index; SCID: Structured clinical interview for DSM disorders; HIPAA: Health Insurance Portability and Accountability Act of 1996.

## Competing interests

The authors declare that they have no competing interests.

## Authors’ contributions

DJB was Co-Principal Investigator and EM was Principal Investigator for the pilot registry. DJB and EM were responsible for designing the study, developing the study protocol, monitoring protocol implementation, and planning data analysis. RD, CN, LL, SR, and JK were involved in the study protocol implementation, day-to-day administration of study protocol, and data/specimen collection. JFJ and RB assisted in study design and development of study protocol, reviewed medical data, assessed eligibility criteria, and along with JSL, classified study subjects. JSL and ERU were involved in study design and review of the study protocol. JSL and HT were responsible for database development and administration. All authors were involved in the preparation of this paper and approved the final manuscript.

## Supplementary Material

Additional file 1: Table S1 Registry provider categories. Click here for file

Additional file 2: Figure S1 Flow chart of healthcare provider recruitment and patient enrollment. Click here for file
