# Supplementary material for: A pilot registry of unexplained fatiguing illnesses and chronic fatigue syndrome
Source: BMC Res Notes. 2013 Aug 2;6:309. doi: 10.1186/1756-0500-6-309 (PMC3750716; doi:10.1186/1756-0500-6-309)
Supplement: Additional file 1: Table S1 — Registry provider categories. [file 1756-0500-6-309-S1.pdf]

Additional file 1: Table S1.

Registry provider categories

Provider Category	Included Specialties
**Physicians**	
Family/General Practice	Family Practice
	Family General Practice
	General Practice
Internal Medicine	Internal Medicine
	Internal Medicine Nephrology
	Gastroenterology/Internal Medicine
	Cardiology
	Pulmonology
Geriatrics	Family Practice Geriatrics
OB/GYN	Obstetrics/Gynecology
Anesthesiology	Anesthesiology
Pain Medicine	Pain Medicine
Pediatrics	Pediatrics
	Pediatrics/Internal Medicine
Allergy/Immunology	Allergy/Immunology
Infectious Disease	Infectious Disease
Rheumatology	Rheumatology
Endocrinology	Endocrinology
Gastroenterology	Gastroenterology
Neurology	Neurology
	Neurosurgery
General surgery	General surgery
	Urology
Orthopedics	Orthopedics
Otolaryngology	Otolaryngology
Ophthalmology	Ophthalmology
Psychiatry	Psychiatry
	Psychiatry/Geriatric
	Psychiatry/Neurology
	Psychiatry/Forensic
Pediatric Specialists	Psychiatry/Pediatric
	Neurology/Pediatric
	Gastroenterology/Pediatric
	Endocrinology/Pediatric
	Infectious Disease/Pediatric

	Pediatrics/Neonatal
Adolescent Medicine	Adolescent Medicine
**Non-Physicians**	
Psychologist	Psychologist
Social Worker	Social Worker
Chiropractor	Chiropractor
Physical Therapist	Physical Therapist
Massage Therapist	Massage Therapist
Acupuncturist	Acupuncturist
Registered Nurse	Registered Nurse
Advanced Practice NP	Advanced Practice NP
Physician Assistant	Physician Assistant
Podiatrist	Podiatrist
Licensed Dietician	Licensed Dietician
Dentist	Dentist
