# Supplementary material for: A pilot registry of unexplained fatiguing illnesses and chronic fatigue syndrome
Source: BMC Res Notes. 2013 Aug 2;6:309. doi: 10.1186/1756-0500-6-309 (PMC3750716; doi:10.1186/1756-0500-6-309)

Additional file 2: Figure S1.

Figure 1 Flow chart of healthcare provider recruitment and patient enrollment

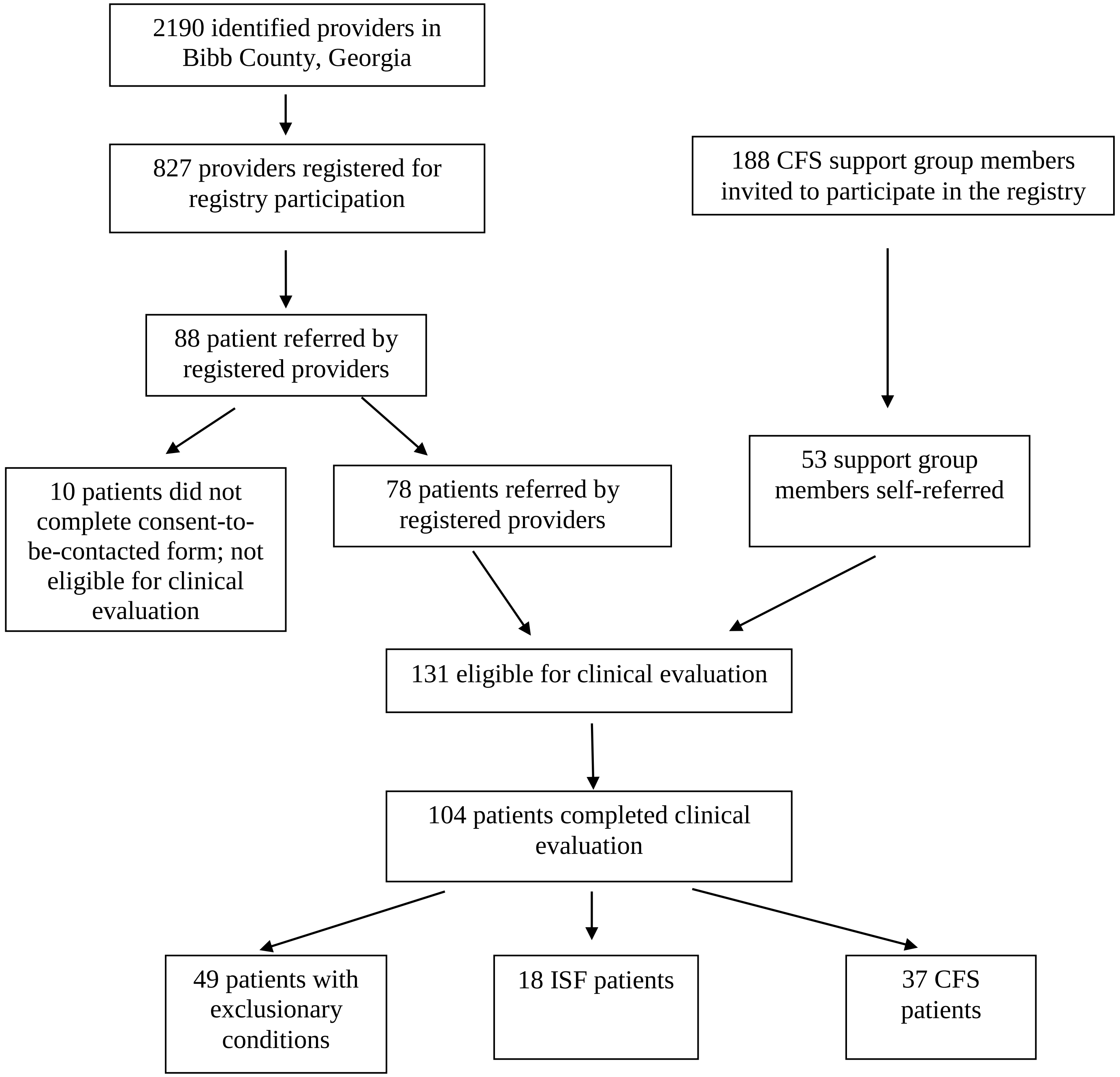

Supplement: Additional file 2: Figure S1 — Flow chart of healthcare provider recruitment and patient enrollment. [file 1756-0500-6-309-S2.pdf]
